# A Facile Approach of Fabricating Electrically Conductive Knitted Fabrics Using Graphene Oxide and Textile-Based Waste Material

**DOI:** 10.3390/polym13173003

**Published:** 2021-09-04

**Authors:** Md Abdullah Al Faruque, Alper Kiziltas, Deborah Mielewski, Maryam Naebe

**Affiliations:** 1 Institute for Frontier Materials (IFM), Deakin University, Geelong, VIC 3216, Australia; m.alfaruque@deakin.edu.au; 2 Research and Innovation Center, Ford Motor Company, Dearborn, MI 48121, USA; akizilt1@ford.com (A.K.); dmielews@ford.com (D.M.)

**Keywords:** recycling waste wool, knitted fabrics, graphene oxide, chemical reduction, electrical conductivity

## Abstract

This research investigated a feasible approach to fabricating electrically conductive knitted fabrics using previously wet-spun wool/polyacrylonitrile (PAN) composite fibre. In the production of the composite fibre, waste wool fibres and PAN were used, whereby both the control PAN (100% PAN) and wool/PAN composite fibres (25% wool) were knitted into fabrics. The knitted fabrics were coated with graphene oxide (GO) using the brushing and drying technique and then chemically reduced using hydrazine to introduce the electrical conductivity. The morphological study showed the presence of GO sheets wrinkles on the coated fabrics and their absence on reduced fabrics, which supports successful coating and a reduction of GO. This was further confirmed by the colour change properties of the fabrics. The colour strength (K/S) of the reduced control PAN and wool/PAN fabrics increased by ~410% and ~270%, and the lightness (L*) decreased ~65% and ~71%, respectively, compared to their pristine fabrics. The Fourier transform infrared spectroscopy showed the presence and absence of the GO functional groups along with the PAN and amide groups in the GO-coated and reduced fabrics. Similarly, the X-ray diffraction analysis exhibited a typical 2θ peak at 10⁰ that represents the existence of GO, which was demolished after the reduction process. Moreover, the wool/PAN/reduced GO knitted fabrics showed higher electrical conductivity (~1.67 S/cm) compared to the control PAN/reduced GO knitted fabrics (~0.35 S/cm). This study shows the potential of fabricating electrically conductive fabrics using waste wool fibres and graphene that can be used in different application fields.

## 1. Introduction

The electrically conductive fabrics, also known as smart fabrics, are currently receiving more attention from both industrial and academic research communities due to their potential application in wearable textiles, supercapacitors, actuators, batteries, electronic displays, solar panels, sensors, health monitoring systems, and the automobile industry [1,2,3]. To impart the electrical conductivity into fabrics, up until now, several approaches have been followed, such as the insertion of metallic wires into fabrics, chemical metallisation and galvanisation of fibres, fabrication of textile fibres with the piezoelectric materials, and even the application of conductive polymer films onto the fabric surface [4,5]. However, these approaches exhibit some major drawbacks, such as the utilisation of toxic and corrosive chemicals, increased fabric weight, formation of additional gaps between the yarns that result in lower dimensional stability of the fabrics, reducing the wearability, flexibility, stretchability, and comfort of the garment [2,6].

Although to suppress these demerits, some reports have been published mentioning the use of flexible conductive polymeric wires rather than metallic wires, suffering from higher electrical resistance and lower conductivity [6]. To avoid this deteriorated electrical conductivity, the insertion or coating of the textile fabrics with the carbon nanotubes (CNTs) has fascinated researchers because of its distinctive physical and chemical characteristics, outstanding electrical and thermal conductivity, superior flexibility, and light weight [7,8]. Therefore, this material is used in diverse applications such as sensors, supercapacitors, energy harvesting and storage, and as cathodes [7,8,9,10]. However, the materials fabricated with CNTs are accountable for creating several critical problems related to human health and safety because of their toxic nature [8]. Hence, it is essential to find a suitable method of making electrically conductive fabrics using a cost-effective, compatible, and eco-friendly approach to fulfil its demand for diverse application areas. The adhesion of graphene sheets onto the fabric surface might be a better idea for tackling the problems discussed earlier. Recently, it has been reported that the use of graphene into composite materials can ensure environmental sustainability, as it increases the physical properties of the host material and lowers the inclusion of other materials into that composite to perform the same function [11]. Besides, graphene can help in reducing the carbon footprint. For example, the addition of only 0.03% (by weight) graphene with concrete can deteriorate 25–33% carbon footprint and 2% global carbon emissions [11]. 

Textile fabrics have a greater surface area, with tremendous flexibility, stretchability, and mechanical properties, that can function as a substrate for depositing graphene oxide [12,13]. Furthermore, it might be possible that the available chemical functional groups in fabrics can be physically or chemically bonded with the functional groups present in GO to ensure proper adhesion of GO with the fabrics [7,8]. The application of graphene oxide (GO) or reduced graphene oxide (rGO) onto the fabric surface using the “brushing and drying” or “dip and dry” technique is a common approach to fabricate electrically conductive fabrics. In one study, the “dip and dry” technique was used to deposit GO onto cotton fabric, and then the GO-coated cotton fabric was passed through the chemical reduction process to introduce electrical properties so that the fabrics can be used in smart and E-textiles based applications [14]. Javed et al. reported the fabrication of graphene-based electro-conductive fabrics obtained through ultraviolet curing methods that can be used as smart textiles in several areas [15]. Recently, Stempien et al. demonstrated the deposition of reduced graphene oxide (rGO) layers on the fabric surface that can be used as a supercapacitor using reactive inkjet printing [16]. Samanta and Bordes reported a uniform deposition of graphene particles onto a polyester (PET) fabric surface and then a reduction of the GO coated fabric with thermal and chemical methods to prepare electrically conductive PET fabrics [17]. Wu et al. reported the production of GO coated viscose knitted fabrics with electrical conductivity suitable for possible applications in conductive devices, smart textiles, and water treatment systems [18]. 

Although commercially available fabrics are mainly purchased and coated with graphene, then reduced by following the physical or chemical approaches to fabricate the electro-conductive fabrics, there are few, if any, reports on the production of knitted fabrics utilising the waste textile sources and their subsequent transformation into electrically conductive fabrics. We have previously reported a successful and eco-friendly transformation of short and non-spinnable waste wool fibres into powder, then fabricated them into the wet-spun wool/PAN composite fibres [19]. In this study, we aimed to investigate the feasibility of developing an electrically conductive fabric from this previously wet-spun wool/PAN composite fibre [19]. In this work, the as-prepared wool/PAN composite fibres were converted into knitted fabrics using the knitting technique. Then, fabrics were coated with GO using the “brushing and drying” technique. The GO-coated wool/PAN knitted fabrics were then chemically reduced (using hydrazine monohydrate) to develop the electrically conductive knitted fabrics that can be used in diverse applications. The morphology, chemical structure, crystallinity, and electrical properties of the knitted fabrics were characterised and compared with control fabrics. 

## 2. Materials and Methods

### 2.1. Materials

The control PAN fibres (100% PAN) and wool/PAN composite fibres (25% wool) were produced using the wet spinning technique, as reported previously [19]. Sulfuric acid (H_2_SO_4_, 98%), expandable graphite, potassium permanganate (KMnO_4_), hydrochloric acid (HCl, 32%), hydrogen peroxide (H_2_O_2_, 30%), and hydrazine monohydrate (N_2_H_4_·H_2_O) were purchased from the Sigma-Aldrich, New South Wales, Australia. All the chemicals were of analytical grade and thus used as received.

### 2.2. Preparation of the Knitted Fabrics

The control PAN knitted fabric (CPKF) and wool/PAN knitted fabric (WPKF) were manufactured using the Fiber Analysis Knitter (FAK) 10-gauge circular knitting machine (with cylinder diameter of 10 inches, and total of 336 needles), Lawson Hemphill, Inc. (Swansea, Massachusetts, USA), at Commonwealth Scientific and Industrial Research Organisation (CSIRO), Geelong Waurn Ponds, following the process mentioned earlier [20]. The digital images of the knitted fabrics are shown in the Supplementary Figure S1. Table 1 shows the specifications of the knitted fabrics. 

### 2.3. Synthesis, Coating, and Reduction of Graphene Oxide (GO)

The synthesis of GO was accomplished using the modified Hummers method [19]. The synthesised GO solution (1% (*w*/*v*)) was coated onto the CPKF and WPKF by the “brushing and drying” technique (Supplementary Section S1) [15], and were named CPGOKF and WPGOKF, respectively. The GO-coated knitted fabrics, CPGOKF and WPGOKF, were then reduced following a chemical-reduction approach using hydrazine vapour exposure (Supplementary materials, Section S2) [21] and were named CPrGOKF and WPrGOKF, respectively.

### 2.4. Characterisation Techniques 

The morphology of knitted fabrics was explored with a Jeol NeoScope (JCM-5000, Jeol Australasia Pty Ltd., Frenchs Forest, NSW, Australia) scanning electron microscope (SEM), where the accelerating voltage was kept at 10 kV. Prior to imaging, all the samples were coated with Leica EM ACE600 gold coater (Leica Microsystems Pty Ltd., Macquarie Park, NSW, Australia). The Fourier-transform infrared (FTIR) spectra analysis of knitted fabrics was accomplished under the attenuated total reflectance (ATR) mode using the Vertex 70 (Bruker Optik GmbH, Rudolf-Prank-Strabe, Ettlingen, Germany) where the scan resolution was 4 cm^−1^, and OPUS 5.5 software was used to perform the baseline correction. The diffraction pattern analysis of the fabric samples was performed at ambient temperature using the X-ray diffraction (XRD) technique (X’Pert Powder, PANalytical, Almelo, Netherlands). During the experiments, both the operating voltage (40 kV) and current flow (30 mA) remained constant. The colour properties (colour strength and CIELAB colour coordinates L*, a*, b*) of all the knitted fabrics were measured by spectrophotometer SF600 Plus-CT (Datacolor, Lawrenceville, New Jersey, USA), keeping the illuminant and observer angle constant (D65 and 10⁰, respectively). The colour strength (K/S) was determined from the light reflectance data according to the Kubelka–Munk equation (Equation (S1), Supplementary materials). Each sample was measured five times and an average value was reported. Standard deviation was not considered due to the minor variation in the colour coordinate values. The electrical surface resistance of the GO-coated and reduced knitted fabrics was measured using the four-point probe technique utilising a digital multimeter (Keysight, model 34461A, Keysight Technologies Australia Pty Ltd Mulgrave, Victoria, Australia). The unit of the surface resistivity is ohm (Ω). Although the surface resistivity is often expressed also in Ohm/square, this is not a valid unit from the dimensional analysis point of view [22]. The resistance of the samples was assessed 20 times and an average value was used to determine the electrical conductivity of the knitted fabrics via the formula shown in Equation (1) [19,23,24,25].

(1) $$\sigma =\;\frac{L}{RA}$$

where *σ* is the conductivity (S/cm), *R* is the resistivity (Ω), *A* is the cross-sectional area of the fabric, which was measured using the width (cm) and thickness (cm) of the fabric samples, and *L* is the length of the fabric samples (cm). 

## 3. Results

### 3.1. Morphology of the Knitted Fabrics

**Figure 1:**
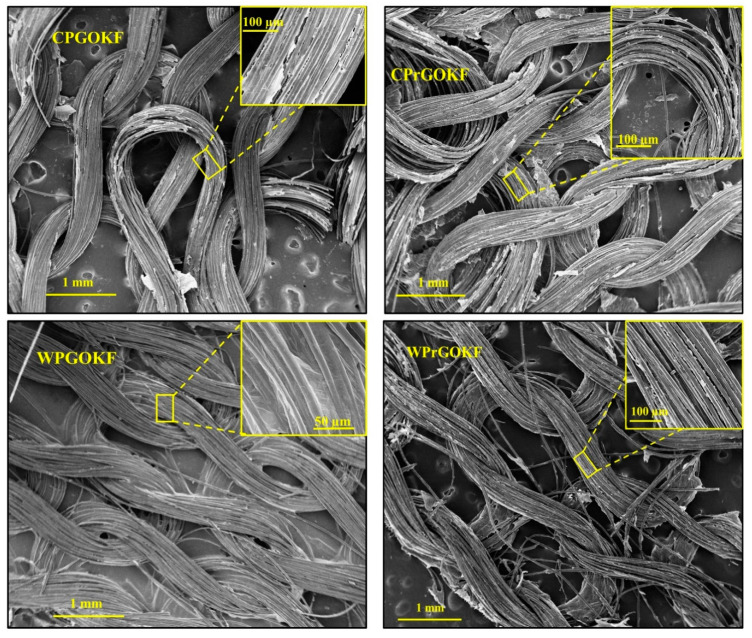
SEM images of control PAN/GO knitted fabric (CPGOKF), control PAN/reduced GO knitted fabric (CPrGOKF), wool/PAN/GO knitted fabric (WPGOKF), and wool/PAN/reduced GO knitted fabric (WPrGOKF).

The surface morphology of GO-coated, and reduced fabric samples is shown in Figure 1 (the morphology of the untreated fabrics is shown in Supplementary Figure S2). The characteristic looping structure of the knitted fabrics was observed from all the SEM images. Unlike untreated fabric samples, both the control PAN/GO knitted fabric (CPGOKF) and wool/PAN/GO knitted fabric (WPGOKF) exhibited the presence of graphene sheets on the fabric surface, confirming the effective incorporation of GO achieved using the “brushing and drying” process [18,26]. Additionally, the wrinkles produced from the GO sheet are observed on the CPGOKF and WPGOKF surface that proved the exfoliation of expandable graphite into graphene and the existence of oxygen-containing functional groups, as reported in our previous study [19]. Moreover, it can be seen that GO was integrated into the interlacing points of the knitted fabrics, which confirms the uniform distribution of the GO sheet that could be effective in enhancing the electrical conductivity of the reduced knitted fabrics (discussed later). An effective reduction of the GO-coated knitted fabrics following the chemical approach could also be confirmed via the SEM images of the reduced fabrics. It can be observed that the wrinkles have been removed from the fibre surface and a few clusters of graphene sheet can be found, which eventually ensured the effective chemical reduction of the knitted fabrics, as reported in previous studies [19,27,28]. In general, the wrinkles on the GO sheets are formed during the fabrication process of GO and these disappeared due to the removal of the water molecules and some oxygen-containing functional groups from the GO sheets [29,30] after reduction. 

### 3.2. FTIR and XRD Analysis of the Knitted Fabrics

**Figure 2:**
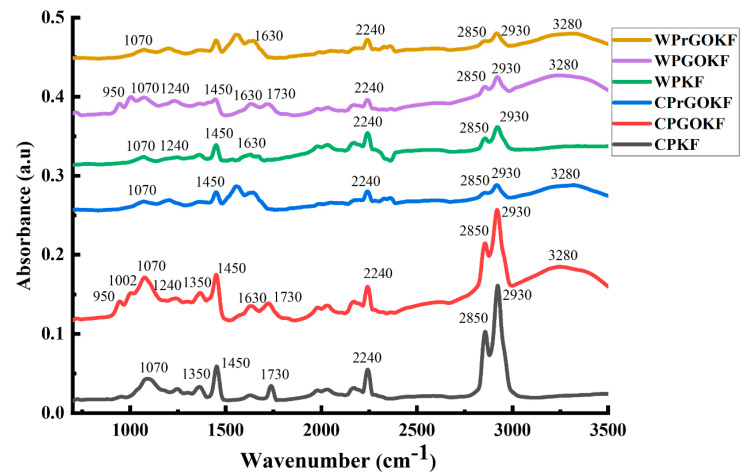
FTIR analysis of control PAN knitted fabric (CPKF), control PAN/GO knitted fabric (CPGOKF), control PAN/reduced GO knitted fabric (CPrGOKF), wool/PAN knitted fabric (WPKF), wool/PAN/GO knitted fabric (WPGOKF), and wool/PAN/reduced GO knitted fabric (WPrGOKF).

The effective linkage of GO with the knitted fabrics was investigated using FTIR analysis as shown in Figure 2 (FTIR spectra of control PAN, wool powder, GO and rGO has been presented in Supplementary Figure S3). In general, wool materials illustrated their distinctive functional peaks at 1240 cm^−1^, 1530 cm^−1^, 1630 cm^−1,^ 2850 cm^−1^, 2930 cm^−1^, and 3280 cm^−1^ which represents the existence of amide III, amide II, amide I, symmetric and asymmetric C-H stretching of the methylene group, and amide A (N-H stretching and O-H stretching), respectively (Supplementary Figure S3a) [31,32,33]. The control PAN signified its functional peak at 2240 cm^−1^, 1470 cm^−1^, and 2930 cm^−1^ which are ascribed to the C≡N (nitrile group), bending and stretching vibration of methylene groups, correspondingly (Supplementary Figure S3a) [34]. The other functional groups have been found at 1070 cm^−1^, 1350 cm^−1,^ and 1730 cm^−1^ due to the C-CN groups, stretching of C-H in CH groups, stretching and absorption of C=O in COOH groups, respectively (Supplementary Figure S3a) [35,36,37,38]. On the other hand, the typical peaks of GO can be seen from 950 cm^−1^ to 1450 cm^−1^, derived from the occurrence of C–O bonds of epoxy groups, hydroxyl groups, and other C-OH vibrations of carboxyl groups, with peaks at 1628 cm^−1^, 1730 cm^−1^, and 3280 cm^−1^ confirming the presence of C=C, C=O, and -OH groups, correspondingly (Supplementary Figure S3b) [19,39]. Nevertheless, after chemical reduction using hydrazine vapour, it can be found that the oxygen-containing functional groups such as carboxyl, alkoxy and epoxy functional groups have diminished (Supplementary Figure S3b) [40]. As GO is composed of different functional groups, such as carboxyl, hydroxyl, carbonyl, and epoxy, which make this material hydrophilic in nature and easily dispersed in water, it can be expected that GO will create strong covalent bonds and hydrogen bonds with the functional groups present in PAN and wool, as found previously [19].

From Figure 2, it is evident that, in the CPKF and CPGOKF, the identical peaks of both PAN and GO are present between 950 cm^−1^ and 3280 cm^−1^, as mentioned earlier, without destroying their functional groups. Besides, after the chemical reduction process, it can be found that the oxygen-containing functional groups have been removed from the CPrGOKF as expected and previously found in the reduction of pure GO materials (Supplementary Figure S3b) [21]. In the case of the WPKF, it is apparent that only amide-III and amide-I (at 1240 cm^−1^ and 1630 cm^−1^) groups are present along with the control PAN peaks, which is due to the chemical dissolution of other protein peaks of wool during the fabrication of wool/PAN composite fibre, as discussed in our previous study [19]. However, WPGOKF and WPrGOKF demonstrated the efficient addition and chemical reduction of GO, similar to the CPGOKF and CPrGOKF. During the incorporation of GO upon the fabric surface, the functional groups of GO and wool/PAN (fabric) interact with each other to form the GO-coated fabrics. At this time, mainly the electrostatic interaction, van der Waals forces, and hydrogen bonds are created [26]. Hence, this FTIR study revealed that the use of the “brushing and drying” technique to incorporate GO into the CPKF and WPKF does not contain any negative impacts on the characteristic chemical groups of PAN and wool. 

**Figure 3:**
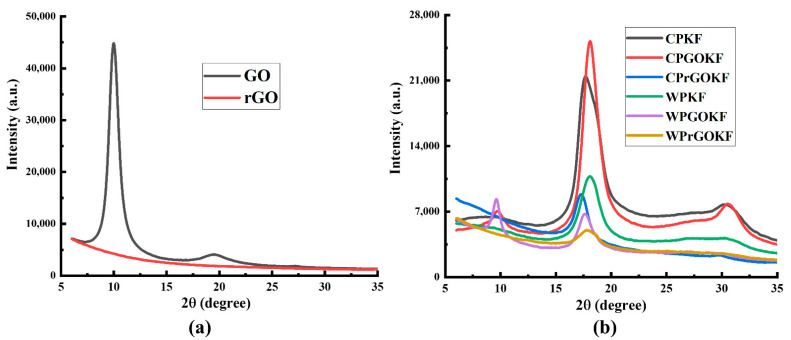
Diffraction pattern analysis of (**a**) graphene oxide (GO) and reduced graphene oxide (rGO); (**b**) control PAN knitted fabric (CPKF), control PAN/GO knitted fabric (CPGOKF), control PAN/reduced GO knitted fabric (CPrGOKF), wool/PAN knitted fabric (WPKF), wool/PAN/GO knitted fabric (WPGOKF), and wool/PAN/reduced GO knitted fabric (WPrGOKF).

To further confirm the inclusion and reduction of GO onto the fabric surfaces as found by the FTIR analysis, the XRD analysis of GO, rGO, and knitted fabrics was performed as shown in Figure 3. A usual intense 2θ peak was found at 10⁰ with a subsequent d-spacing of 0.8 nm, which ensures the presence of stacked oxygen atoms among the graphene sheets (Figure 3a) [19,41]. Besides, the other weaker 2θ peak at around 18.5⁰ with a consecutive d-spacing of 0.4 nm indicates that a small portion of GO might not be entirely intercalated by the oxygen-containing functional groups [41]. However, after the chemical reduction process, both the strong and weak 2θ peaks diminished, as found previously [19]. In the case of the CPKF and WPKF, it can be found that both fabrics showed an identical primary 2θ peak at 17.5⁰ and secondary 2θ peak at 30.5⁰, resembling the (100) and (110) crystallographic planes of the pristine PAN [19,42,43]. The presence of a sharp 2θ peak at 10⁰ along with the other 2θ peaks at 17.5⁰ and 30.5⁰ is visible in the case of the CPGOKF and WPGOKF, which proves the accumulation of GO sheets onto the knitted fabrics. Similarly, after accomplishing the GO-reduction process, the removal of the primary 2θ peak at 10⁰ can be realised in the case of the CPrGOKF and WPrGOKF without hampering their original 2θ peaks at 17.5⁰ and 30.5⁰, respectively. However, the intensity of these peak positions decreased due to the chemical reduction of the fabrics. This might be due to the transformation of the original structure of the pristine PAN towards the aromatic ring or ladder structures, supporting the stabilisation of PAN, which was also reported in the previous studies [43,44]. Therefore, the XRD analysis of all the knitted fabrics confirms the incorporation of GO and its subsequent effective chemical reduction, which also support the findings of the FTIR. 

### 3.3. Colour Change Properties of the Knitted Fabrics

**Figure 4:**
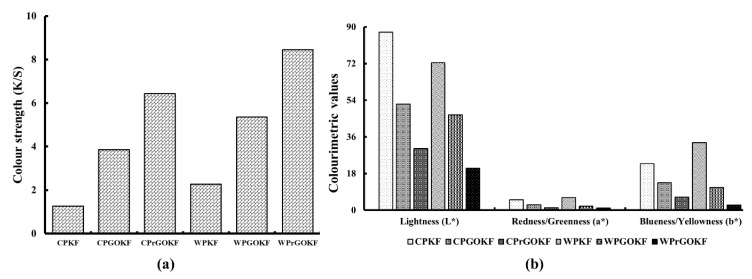
(**a**) Colour strength and (**b**) Colourimetric values of the control PAN knitted fabric (CPKF), control PAN/GO knitted fabric (CPGOKF), control PAN/reduced GO knitted fabric (CPrGOKF), wool/PAN knitted fabric (WPGO), wool/PAN/GO knitted fabric (WPGOKF), and wool/PAN/reduced GO knitted fabric (WPrGOKF).

Effective incorporation and reduction of graphene sheets onto the fabric surface can change the colour of the fabrics [45,46]. Therefore, the colour strength (K/S) and CIELAB colour coordinates L*, a*, b* (Supplementary Figure S4) of untreated and treated fabrics were evaluated and are illustrated in Figure 4a,b). Figure 4a shows that the K/S of the untreated fabrics was the lowest and with the addition and then reduction of GO, the colour strength of the fabrics increased. This can confirm that GO has been introduced onto the fabric surface and GO reduction has changed the surface of the fabrics. Subsequently, light reflectance and scattering have changed, resulted in changes in fabric colour. The fabrics produced with the wool/PAN composite fibre showed higher colour strength compared to the fabrics knitted with the control PAN fibres, which might be due to the presence of wool in the composite fibre that eventually created an excellent covalent bond with GO. The hydroxyl groups of GO showed strong affinity towards the groups present in the wool/PAN composites that resulted in higher absorption of GO onto the wool/PAN composite fabrics. On the other hand, as PAN is hydrophobic, it showed a lower affinity with GO that resulted in a lower proportion of GO uptake and ultimately lower colour strength than the wool/PAN fabrics. 

The CIELAB colour coordinates also indicate the increment and decrement of colour of any material. Generally, a higher value of L* [0 (black) ≤ L* ≤ 100 (white)] means greater lightness of the shade. Higher a* indicated greater redness (if a* > 0) or lesser greenness (if a* < 0), higher b* indicates greater yellowness (if b* > 0) or lesser blueness (if b* < 0) of the shade [47]. From Figure 4b, it can be observed that the lightness (L*) of the control PAN knitted fabric decreased by about one-third with the incorporation of GO and its subsequent reduction. A similar tendency was found in the wool/PAN knitted fabric where the lightness (L*) reduced from ~72 to ~20, respectively. This reduction in lightness of the fabric samples demonstrated that with the introduction of GO, the colour of the fabrics changed from their original light (white or ecru) colour to the darker (black) colour. Similarly, the redness/greenness (a*) and blueness/yellowness (b*) values were also found to decrease with the addition and reduction of GO, which also supports our findings of the increasing depth of colour among the samples. As the WPrGOKF showed a lower lightness (L*) value than CPrGOKF, it might be possible that the WPrGOKF exhibits higher electrical conductivity than CPrGOKF. 

### 3.4. Electrical Conductivity of the Knitted Fabrics

The four-point probe method was used to further investigate the effective reduction of GO and examine the electrical properties of knitted fabrics. For both GO-coated PAN and wool/PAN knitted fabrics (CPGOKF and WPGOKF), neither indicated any electrical conductivity nor any surface resistance (data shown as “Overloaded”), which was anticipated as GO is an electrically insulating material, as discussed earlier [19]. Nonetheless, upon accomplishing the chemical reduction via hydrazine monohydrate, both the reduced GO-knitted fabrics (i.e., CPrGOKF and WPrGOKF) demonstrated surface resistivity and electrical conductivity. The surface resistivity of CPrGOKF and WPrGOKF was found to be ~285 ± 1.72 Ω and ~50 ± 1.15 Ω and the electrical conductivity was ~0.35 ± 0.78 S/cm and ~1.67 ± 0.83 S/cm, respectively. This superior electrical conductivity of WPrGOKF over CPrGOKF is attributed to the effective linkage of the amino groups of wool with the carboxyl and hydroxyl groups of the GO sheets [19]. This can also be evident from the colour properties study of the knitted fabrics where the colour strength (K/S) was higher and the lightness (L*) value was lower in the case of WPrGOKF compared with CPrGOKF. The higher K/S and lower L* represent the effective reduction of GO that changed the colour of the sample into a darker shade from the lighter shade [15,25]. Furthermore, several authors reported that reduced wool/graphene composites exhibited higher electrical conductivity than silk/graphene, cotton/graphene, and flax/graphene composites, which might be due to the effective chemical bonding among various functional groups of GO and wool [15,48,49]. The electrical conductivity of fabricated WPrGOKF produced in this study was found to be superior to some other reported graphene incorporated fabrics, as shown in Table 2. This higher electrical conductivity of WPrGOKF further proves the efficiency of coating GO on the fabric surface following the brushing and drying technique, and the use of hydrazine as a reducing agent. Considering the higher electrical properties of WPrGOKF, it can be predicted that it has an excellent potential to be applied in numerous application areas, such as actuators, sensors, supercapacitors, electronic conductors, and wearable textiles [50,51]. 

## 4. Conclusions

This study investigated a facile approach to producing an electrically conductive knitted fabric utilising natural fibre waste that can be applied in diverse application areas. The fabrics were knitted using wet-spun control PAN and wool/PAN composite fibres. The fabricated knitted fabrics were then coated with graphene oxide (GO) using a simple and feasible process. The GO-coated fabrics underwent a chemical reduction process to impart the electrical conductivity. The morphological study demonstrated the effective coating and reduction of graphene oxide on the knitted fabrics. The FTIR analysis revealed the presence of all the characteristic functional groups of wool, PAN, and GO while confirming the removal of the oxygen-containing functional groups upon the reduction of GO. XRD analysis showed the presence of a typical 2θ peak at 10⁰ that resembles graphene, which was demolished during the reduction process. The colour properties study exhibited the increased trend of colour strength (K/S) with the incorporation of GO onto the fabric surface and it was the highest in the case of the reduced fabric samples, which evidently supports the efficient chemical reduction of the fabrics. The wool/PAN/reduced GO knitted fabrics indicated higher colour strength and lower lightness (L*) compared to the control PAN/reduced GO knitted fabrics, which might be due to the presence of the amino and hydroxyl groups in the wool/PAN composite fabrics that increased the affinity between the wool/PAN knitted fabrics and GO compared to the control PAN knitted fabrics and GO. From the electrical conductivity study, it was evident that the wool/PAN/reduced GO knitted fabrics demonstrated lower resistance (50 Ω) and higher conductivity (1.67 S/cm) than the control PAN/reduced GO knitted fabrics (285 Ω and 0.35 S/cm, respectively), which might be due to the adhesion and reduction of a greater portion of GO on the wool/PAN knitted fabrics than the control PAN knitted fabrics. Besides, this conductivity is much higher than some other GO-coated and reduced fabrics reported earlier. Therefore, it can be concluded that the electrically conductive wool/PAN/reduced GO knitted fabric fabricated in this study using waste wool fibres and a simple but feasible approach has the potential for application in several areas, including automobiles as a conductive fabric to reduce the use of metals, especially in the car seat, actuators, sensors, supercapacitors, electronic conductors, and wearable textiles. 

## Figures and Tables

**Table 1:** Specification of the knitted fabrics produced in this study.

Sample	Course Per Inch (CPI)	Wales Per Inch (WPI)	GSM (g/m^2^)	Loop Length (mm)	Yarn Diameter (µm)	Yarn Count (tex)
CPKF	16	18	108	2.90	17	20
WPKF	18	22	116	2.90	20	26

**Table 2:** Electrical conductivity of graphene incorporated textile fabrics found in literature.

Fabric Sample	Reduction Process	Resistance (Ω)	Conductivity (S/cm)	References
Polyester/graphene (woven)	Thermal reduction (200 °C, 2 h)	-	1.28 × 10^−5^	[17]
Polyester/graphene (woven)	Chemical reduction (L-ascorbic acid)	-	8.3 × 10^−6^	[17]
Cotton/graphene (knitted)	Conductive graphene with SDBS * surfactants	-	0.359	[48]
Flax/graphene (knitted)	Conductive graphene with SDBS * surfactants	-	0.040	[48]
Wool/graphene (knitted)	Conductive graphene with SDBS * surfactants	-	0.426	[48]
Cotton/graphene (woven)	Chemical reduction (hydrazine)	1,070,000	2.1 × 10^−5^	[25]
Silk/graphene (woven)	Thermal reduction (200 °C, 30 min)	130,400	7.89 × 10^−6^	[52]
Glass/graphene nanoplatelets (woven)	-	-	0.0005	[53]
polyacrylonitrile/graphene (knitted)	Chemical reduction (hydrazine)	285	0.35	This study
Wool/PAN/graphene (knitted)	Chemical reduction (hydrazine)	50	1.67	This study

* Sodium dodecyl benzenesulfonate (SDBS).

## Data Availability

The raw/processed data required to reproduce these findings cannot be shared at this time as the data also forms part of an ongoing study.

## Supplementary Materials

The following are available online at https://www.mdpi.com/article/10.3390/polym13173003/s1, Figure S1: Digital images of the control PAN knitted fabric (CPKF) and wool/PAN knitted fabric (WPKF), Figure S2: SEM images of control PAN knitted fabric (CPKF) and wool/PAN knitted fabric (WPKF), Figure S3: FTIR analysis of (a) control PAN and wool powder; (b) pure graphene oxide (GO) and reduced graphene oxide (rGO), Figure S4: Digital images of colour change of the wool/PAN knitted fabric (WPKF), wool/PAN/GO knitted fabric (WPGOKF) and wool/PAN/reduced GO knitted fabric (WPrGOKF) upon the coating and reduction of graphene oxide (GO).
