# Supplementary material for: A Facile Approach of Fabricating Electrically Conductive Knitted Fabrics Using Graphene Oxide and Textile-Based Waste Material
Source: Polymers (Basel). 2021 Sep 4;13(17):3003. doi: 10.3390/polym13173003 (PMC8434548; doi:10.3390/polym13173003)
Supplement: Supplementary file 1 [file polymers-13-03003-s001.zip › polymers-1351150-supplementary.pdf]

Supplementary Materials

Article

A Facile Approach of Fabricating Electrically Conductive Knitted Fabrics Using Graphene Oxide and Textile-Based Waste Material

Authors
Md Abdullah Al Faruque[1], Alper Kiziltas[2], Deborah Mielewski[2] and Maryam Naebe[1]*

Affiliations
[1]Deakin University, Institute for Frontier Materials (IFM), Geelong, Victoria 3216, Australia
[2]Research and Innovation Center, Ford Motor Company, Dearborn, 48121, Michigan, USA

*** Corresponding author:**
E-mail: maryam.naebe@deakin.edu.au (Maryam Naebe)

Section S1: Preparation of the Graphene oxide (GO) coated knitted fabrics

The GO solution (1% (w/v)) was applied onto the control PAN knitted fabric (CPKF) and wool/PAN knitted fabric (WPKF) utilising the "brushing and drying" technique [1, 2]. In brief, both the CPKF and WPKF were placed on a suitable platter, firmly cemented with an adhesive tape, and the GO solution was brushed over the fabrics. The fabrics were then dried at ambient temperature for 8 to 10 hrs in the fume hood. This brushing and drying process was replicated 3 times to get GO-coated knitted fabrics. These fabrics were named as Control PAN/GO knitted fabric (CPGOKF) and wool/PAN/GO knitted fabric (WPGOKF).

Section S2: Reduction of the Graphene oxide (GO) coated knitted fabrics

By following a chemical-reduction approach, all the GO-coated knitted fabrics (e.g., CPGOKF and WPGOKF) were reduced using the hydrazine monohydrate vapour exposure [3]. Briefly, nearly 7 cm of CPGOKF and WPGOKF was placed on a microscopic slide and implanted into

a flask. 2-3 ml of hydrazine monohydrate was streamed into the flask and heated at 80 °C for 24 hrs to perform the chemical reduction of the fabrics using the hydrazine vapour inside the chamber [3-5]. The reduced knitted fabrics were then renamed as Control PAN/reduced GO knitted fabric (CPrGOKF) and wool/PAN/reduced GO knitted fabric (WPrGOKF), respectively.

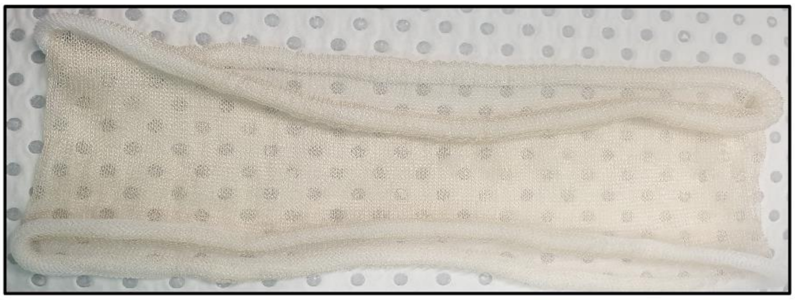

CPKF

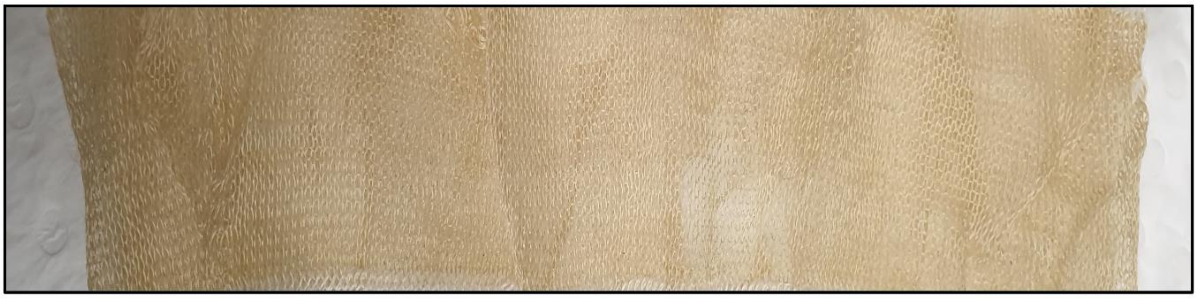

WPKF

Figure S1. Digital images of the control PAN knitted fabric (CPKF) and wool/PAN knitted fabric (WPKF).

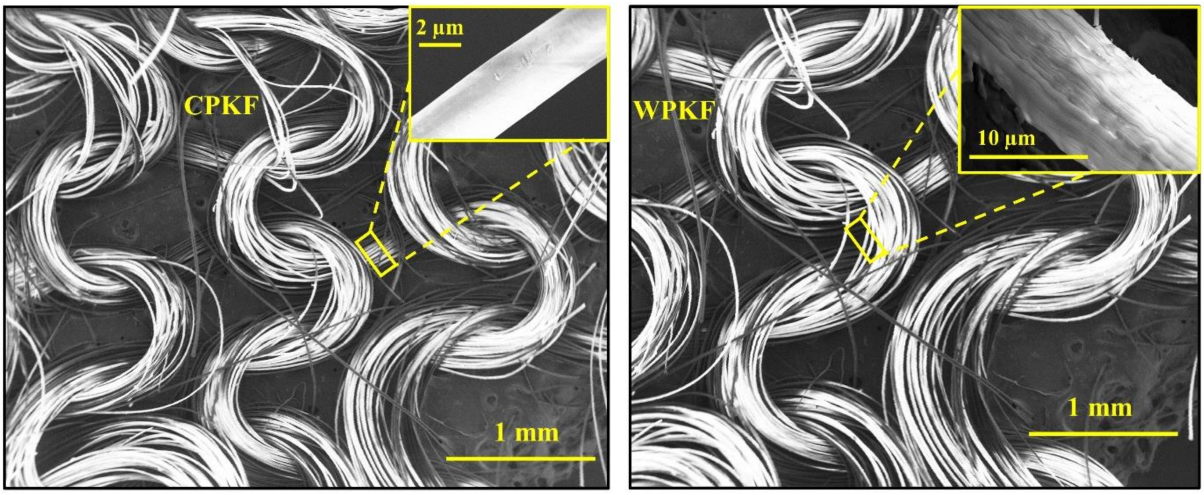

Figure S2. SEM images of control PAN knitted fabric (CPKF) and wool/PAN knitted fabric (WPKF).

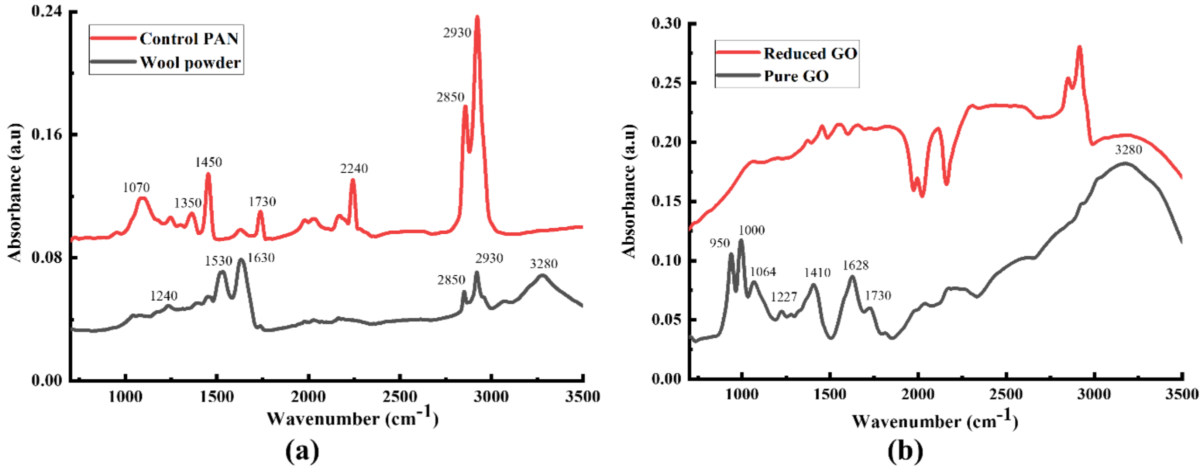

Figure S3. FTIR analysis of (a) control PAN and wool powder; (b) pure graphene oxide (GO) and reduced graphene oxide (rGO).

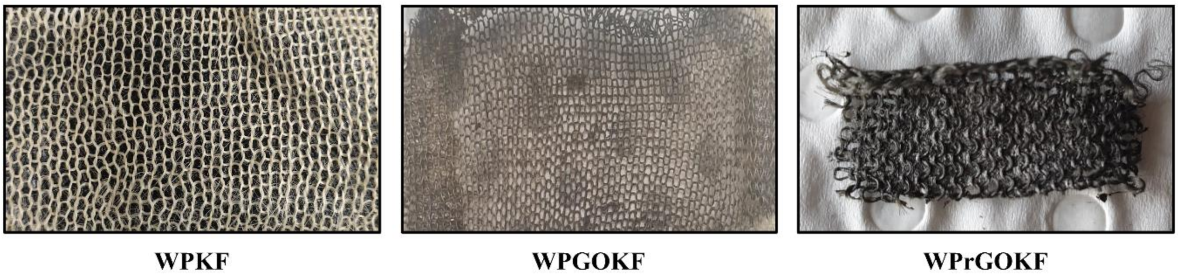

Figure S4. Digital images of colour change of the wool/PAN knitted fabric (WPKF), wool/PAN/GO knitted fabric (WPGOKF) and wool/PAN/reduced GO knitted fabric (WPrGOKF) upon the coating and reduction of graphene oxide (GO).

Equation S1

The colour strength (K/S) was determined from the light reflectance data according to the Kubelka-Munk equation [6]:

$$K/S = (1-R)^2 / 2R \quad \text{(S1)}$$

where K is the absorption coefficient, S is the scattering coefficient, and R is the reflectance.

References

[1] K. Javed, C. Galib, F. Yang, C.-M. Chen, and C. Wang, "A new approach to fabricate graphene electro-conductive networks on natural fibers by ultraviolet curing method," *Synthetic Metals,* vol. 193, pp. 41-47, 2014.

[2] W.-w. Liu, X.-b. Yan, J.-w. Lang, C. Peng, and Q.-j. Xue, "Flexible and conductive nanocomposite electrode based on graphene sheets and cotton cloth for supercapacitor," *Journal of Materials Chemistry,* vol. 22, no. 33, pp. 17245-17253, 2012.

[3] S. Pei and H.-M. Cheng, "The reduction of graphene oxide," *Carbon,* vol. 50, no. 9, pp. 3210-3228, 2012.

[4] M. A. Al Faruque, R. Remadevi, A. Guirguis, A. Kiziltas, D. Mielewski, and M. Naebe, "Graphene oxide incorporated waste wool/PAN hybrid fibres," *Scientific reports,* vol. 11, no. 1, pp. 1-12, 2021.

[5] J. T. Robinson, F. K. Perkins, E. S. Snow, Z. Wei, and P. E. Sheehan, "Reduced graphene oxide molecular sensors," *Nano letters,* vol. 8, no. 10, pp. 3137-3140, 2008.

[6] A. D. Broadbent, *Basic principles of textile coloration*. West Yorkshire, England: Society of Dyers and Colourists, 2001.
